# Catchment Level Water Resource Constraints on UK Policies for Low‐Carbon Energy System Transitions by 2030

**DOI:** 10.1002/gch2.201700006

**Published:** 2017-05-11

**Authors:** D. Dennis Konadu, Richard A. Fenner

**Affiliations:** ^1^ Department of Engineering University of Cambridge Trumpington Street Cambridge CB2 1PZ UK

**Keywords:** carbon budgets, low‐carbon energy, water abstraction, water‐energy nexus

## Abstract

The UK government has proposed different low‐carbon energy system options that lead to meeting its greenhouse gas emissions target of 80% reduction on 1990 levels by 2050. While these energy system options meet emission targets at feasible economic cost, water requirement for the deployment of the proposed energy technology mix is not adequately accounted for. This may become critical, as some of the proposed energy technologies are relatively more water‐intensive, and could result in significant future water resource constraints. Previous studies have analyzed the potential water resource constraints of future energy systems in the UK at national scale. However, water must be considered as a local resource with significant regional variability. This paper uses a linear spatial‐downscaling model to allocate water‐intensive energy system infrastructure/technologies at catchment level, and estimates water requirements for the deployment of these technologies for the Committee on Climate Change Carbon Budgets in 2030. The paper concludes that while national‐scale analysis shows minimal long‐term water related impacts, catchment level appraisal of water resource requirements reveals significant constraints in some locations. This has important implications for regions where the water‐energy nexus must be analyzed at appropriate spatial resolution to capture the full water resource impact of national energy policy.

## 1 Introduction

Meeting both rising global energy and water demand,^1^, ^2^ while reducing greenhouse gas (GHG) emissions to avoid climate change/global warming tendencies (e.g. refs. 3, 4), is one of the pressing global challenges of the 21st century. Furthermore, this must be achieved while ensuring wider environmental sustainability. The energy supply sector constitutes one of the highest GHG emissions sources in both developing and industrialized countries. Thus, meeting this challenge will require a major expansion of energy provision, while simultaneously transitioning away from the current fossil fuel dominated energy system to a low‐carbon regime.

Water and energy provision are intricately interdependent (although some low‐carbon energy technologies are generally water neutral, e.g., solar photo voltaic (PV) and wind power). Water is required across most parts of the energy supply chain, particularly for the following activities: extraction and refining of fossil fuels, hydropower generation, thermal power plant cooling, and increasingly in the irrigation of bioenergy crops. Thus, delivering the required expansion and transition to low‐carbon energy systems will have direct implications for water resource use. Whether these demands for water can be met sustainably needs to be more fully examined and understood. A key factor is how water resource availability varies across geographical regions. This poses significant challenges to the increased deployment of energy technologies, which require water, and is particularly critical for those regions with poor and limited renewable water resources. These constraints are magnified further in highly populated, high energy demanding, and industrialized regions where water demand for energy is projected to increase, vis‐a‐vis demand by other sectors.^5^

In the UK, the Climate Change Act 2008 stipulates an ambitious target of reducing GHG emissions by 80% on 1990 levels by 2050.^6^ The transition to a low‐carbon energy system is considered a top priority, as demonstrated through the Energy Act 2013,^7^ with the rolling out of Electricity Market Reform which has been designed to accelerate the delivery of low‐carbon and renewable energy deployment and climate change targets at lower cost while maintaining reliability.^8^ The main policy blueprint of the UK government's low‐carbon energy system transition agenda are captured in the 2050 Carbon Plan,^9^ and the legally sanctioned five‐yearly Carbon Budgets of the Committee on Climate Change (CCC). These provide scenarios and pathways, which project different mixes of energy supply technologies to 2050. While these policies project the achievement of 80% GHG emissions reduction target at minimal cost to the UK economy, implications of this transition for sustainable environmental resources, principally water use, are not adequately dealt with. This is more important as some of the low‐carbon energy technologies considered in these policy pathways are comparatively highly water intensive. In the UK water resource availability is highly spatially variable, there is therefore a limit on how much thermal generation could be deployed across different regions even though the technology may satisfy low‐carbon targets. Water resource impacts within different regions may be further exacerbated by climate change/variability impacts, as water availability in some UK regions are projected to significantly decrease in coming decades.^10^

The above uncertainties present potential future challenges of simultaneously delivering long‐term low‐carbon energy, and maintaining water resource sustainability in the UK. This has led to several recent studies on the UK water‐energy nexus, most of which focus on thermal electricity generation and impacts of different energy system trajectories and appraising the impacts of technological assumptions and using analysis at different spatial scales. Byers et al.^11^ and Konadu et al.^12^ analyzed the UK Carbon Plan pathways on a national scale, each considering different scenarios of cooling technology deployment and future location of power plants. Both studies concluded that the UK could face significant water resource stress under a future of high carbon capture and storage (CCS) deployment for thermal electricity generation. Using outputs from the Combined Gas and Electricity Network planning model,^13^ Byers et al.^14^ also analyzed the regional water resource requirement across Great Britain. Similarly, Murrant et al.^15^ analyzed water resource implications of thermal generation on a regional administrative scale for 2050 using the Energy Technologies Institute's Energy Systems Modelling Environment pathways.

These studies have provided important insights on future water resource implications of delivering low‐carbon energy in meeting GHG emissions targets. However, no study has yet analyzed the CCC Carbon Budgets, which are the legally sanctioned energy system transition pathways that underlie the UK Government GHG emissions reduction strategy and policy, particularly at the catchment scale. The questions therefore is, how tractable are the carbon Budgets in terms of water resource requirements, particularly at the catchment level? This is more critical since water resource decisions must be considered at the catchment scale, an analysis which no UK study has yet considered, even though some of the above mentioned studies have considered studies at the regional administrative level.^15^ Water is essentially a local or regional resource, and needs to be assessed where it naturally accumulates or is depleted, hence this study focuses on water‐energy nexus implications derived from catchment level water resource accounting. This avoids having to scale or proportionally divide water resource data (as recorded at catchment level) to fit different geographically derived areas. Moreover, should water resource availability provide additional criteria besides cost and GHG reduction, as a basis for pursuing different energy system trajectories to 2050 besides the Carbon Budgets?

This paper delves into these questions with an analysis of the water resource implications of the CCC's Carbon Budget Central scenario to 2030 at the catchment/hydrological region level for England and Wales and at the country level for Scotland and Northern Ireland. The output of this type of analysis will provide a nuanced picture of the UK water‐energy nexus in terms of the spatiality of potential long‐term impacts at an appropriate decision‐making scale (river catchment level), which has currently not been analyzed, and highlights how national energy policy is differently water constrained in different parts of the UK.

The choice of 2030 is predicated on the fact that current government emissions reduction policy expressed in the CCC Carbon Budgets only extends to 2030. Furthermore, analysis beyond 2030 introduces considerable uncertainties driven by the wider assumptions that need to be made. The study uses a linear spatial downscaling model to allocate water intensive future energy system infrastructure/technologies at the basin level, and an integrated resource accounting methodology, which tracks and estimates fresh and tidal water resource requirements for the deployment of these technologies. The output of the water requirement analysis is then compared, first with recent (2015) levels of water resource requirement for energy for each catchment, and then with the overall water resource requirements of the UK Carbon Plan pathways at the national level, to elicit the overall differences in water resource impacts posed by these pathways at these different scales of analysis. The paper concludes by relating insights from the UK study to other jurisdictions where water resource availability could hinder the deployment of low‐carbon energy.

## 2 Results

The results are presented in three parts. First, we present the output of the current (2015) water resource abstraction for power generation and oil refining for each of the catchments (hydrological regions)/countries. This is followed by the results of the spatial downscaling of the 2030 power generation capacity of the CCC Central scenario, together with the associated water requirements across the basins/countries under consideration. We then present a comparison of the water resource requirement in 2030 of the CCC Central scenario and water resource requirements of the four energy system pathways of UK 2050 Carbon Plan—Core MARKAL (cost optimized), high renewables, high nuclear, and high CCS—on a national scale (see Table S5, Supporting Information, for detailed description of these pathways).

### 2.1 Current UK Water Requirements for Thermal Power Generation and Oil Refining

**Figure 1:**
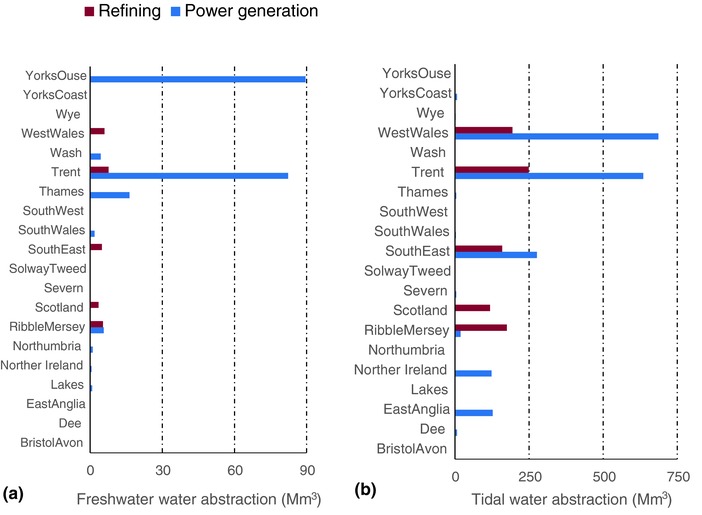
Comparison of current (2015) fresh and tidal water abstraction for power generation and oil refining for different UK river catchments (hydrological regions)/countries: a) Freshwater; b) Tidal water.

**Figure** **1** presents the current licensed water (fresh and tidal only) abstraction requirement for power generation and oil refining by source for the major river catchments (hydrological regions)/countries, and reflect the current distribution of operational thermal power generation and refineries in the UK. The results show the Trent, West Wales, and South East river catchments (hydrological regions) as the highest overall tidal water demand for thermal power generation in the UK. These regions together constituted a little over 84% of tidal water abstractions required for power generation in 2015. The current freshwater required for thermal power generation is predominantly abstracted in the Trent and Yorkshire Ouse catchments, which constitute 40% and 44% of the overall estimated UK demand respectively of the total abstraction requirement in 2015. Water abstraction for oil refining processes is associated with only five basins/countries—West Wales, Trent, South East, Scotland, and Ribble‐Mersey—with the highest estimated demand within the Trent basin.

**Figure 2:**
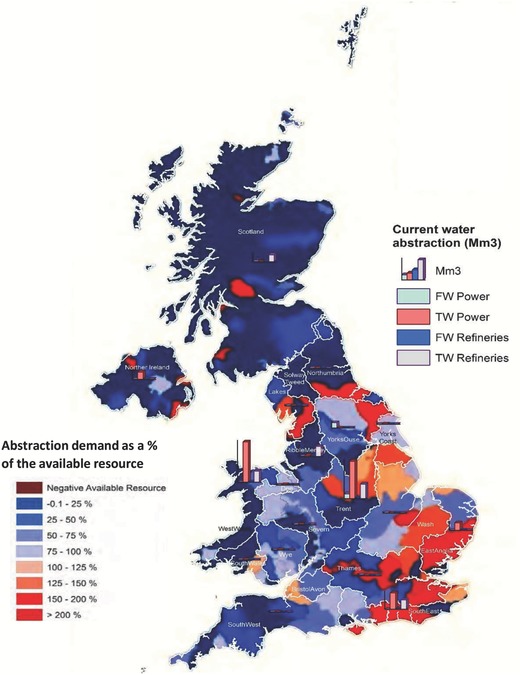
Map showing the spatial distribution of the estimated current water resource (fresh and tidal) demand for power generation and oil refinery for 2015, and the present‐day water resource abstraction demand as a percentage of the available resource at the average of Q95 and 70 low flow conditions (Source: ref. 16: CCRA2: Updated projections for water availability for the UK – Final Report). FW = Freshwater; TW = Tidal water.

**Figure** **2** illustrates the spatial distribution of current levels of water abstraction for power generation and refinery operations for the catchments/countries, and how these relate to the current national variation of the overall abstraction demand versus resource availability based on Climate Change Risk Assessment (CCRA2) updated projections of water availability in the UK.^16^ This shows that with the exception of South East and East Anglia hydrological regions, the overall water resource availability within the major basins/countries that host power stations and refineries in the UK are currently under minimal abstraction stress. Notwithstanding this, some sub‐catchments in most of the major basins across England in particular, exhibit different levels of over‐abstraction (see Figure 2).

### 2.2 Distribution of Power Stations and Refineries and Associated Water Requirement in 2030

**Figure 3:**
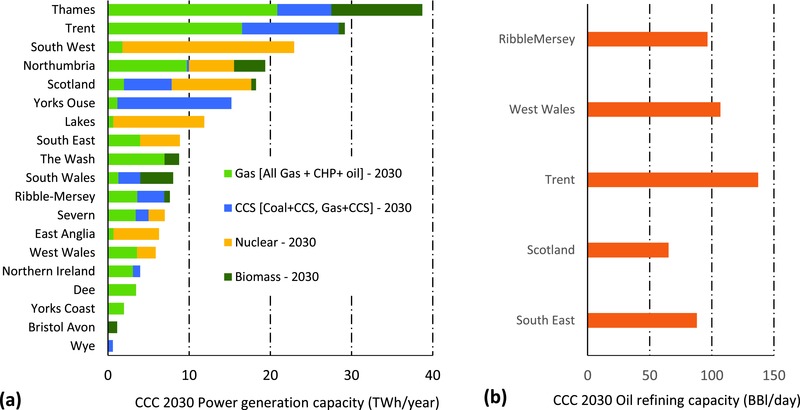
Regionally downscaled (from national to river catchment/country) UK thermal power generation by technology and capacity a) and estimated oil refining capacity b) for the CCC Central scenario in 2030.

The output of the spatial downscaling of power generation and oil refining capacity across the major river catchments (hydrological regions)/countries of the CCC Central scenario is presented in **Figure** **3**. Figure 3a shows the power generation capacity distribution by technology, while Figure 3b presents the estimated daily oil refining capacity distribution of the CCC Central scenario in 2030. The output shows the Thames basin to have the highest projected power generation capacity, followed by the Trent and the South West. The Thames basin is projected to be dominated by Gas and Biomass generation, with Gas and Gas/Coal + CCC dominating the Trent basin. The South West on the other hand is projected to host the highest nuclear generation capacity. Oil refining capacity, however, follows the same distribution pattern as today, with the Trent basin projected to host the highest capacity.

**Figure 4:**
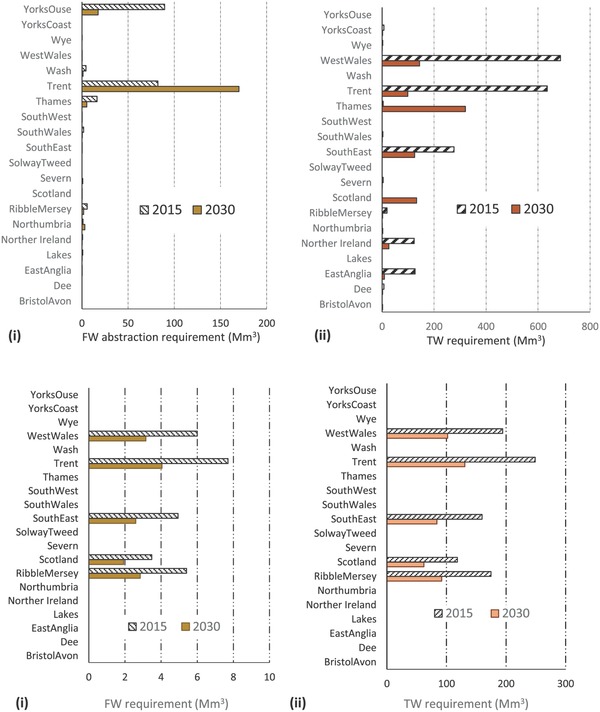
a) Comparison of the current (2015) and 2030 CCC Central scenario water abstraction requirement for UK thermal power generation: (i) Freshwater (FW) and (ii) Tidal water (TW) across major UK river catchments/hydrological regions. b) Comparison of the current (2015) and 2030 CCC Central scenario water abstraction requirement for UK oil refining: (i) Freshwater (FW) and (ii) Tidal water (TW) across major UK river catchments/hydrological regions.

The associated fresh and tidal water requirement for the projected power generation and oil refining capacities in 2030 (Figure 3) compared to current (2015) abstraction levels for each of the catchments/hydrological regions are presented in **Figure** **4**a,b, respectively. The results show that with the exception of the Trent basin which projects a significant increase by 2030, freshwater abstraction for power generation across all other basin/countries are projected to significantly reduce relative to current abstraction levels. Similarly, tidal water abstraction in all major power generation basins/countries, with the exception of the Thames basin and Scotland are projected to significantly reduce.

**Figure 5:**
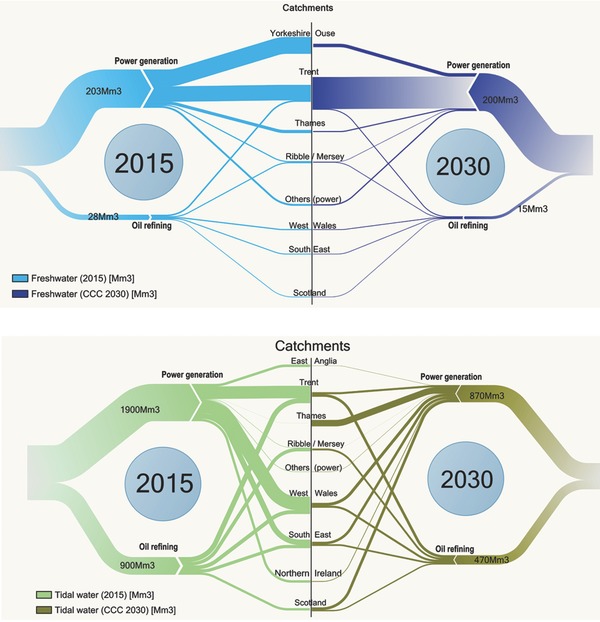
a) A comparison between the estimated current (2015) and 2030 CCC projected overall UK freshwater abstraction for thermal power generation and oil refining (including catchments with significant projected change). b) A comparison between the estimated current (2015) and 2030 CCC projected overall UK tidal water abstraction for thermal power generation and oil refining (including catchments with significant projected change).

**Figure** **5**a,b illustrates the overall change between the current (2015) and 2030 (CCC projections) fresh and tidal water respectively, across the UK (including catchments with significant projected water abstraction change). Overall, freshwater abstraction across the UK reduces marginally (7%) relative to 2015 levels. This is as a result of the sharply contrasting freshwater requirement of the two main power generation basins (with the highest freshwater abstraction in 2015), in the Yorkshire Ouse and the Trent basins. While freshwater requirements in the Trent basin are projected to double (≈106%) in 2030, abstraction requirement of the Yorkshire Ouse is projected to decrease by ≈80% (see Section 3). On the other hand, the overall tidal water abstraction for power generation decreases significantly nationally by 54% in 2030. Abstraction in all major power generation basins/countries, with the exception of the Thames basin and Scotland are projected to significantly reduce. Tidal water abstraction for thermal power generation in the Thames basin and Scotland are however, projected to increase by over 700‐ and 130–fold, respectively relative to 2015 levels. Water abstraction requirement for oil refining in 2030 decreases by 34% across all basins for both fresh and tidal water.

### 2.3 Comparison of Water Requirement CCC Central Scenario and the UK Carbon Plan in 2030

**Figure 6:**
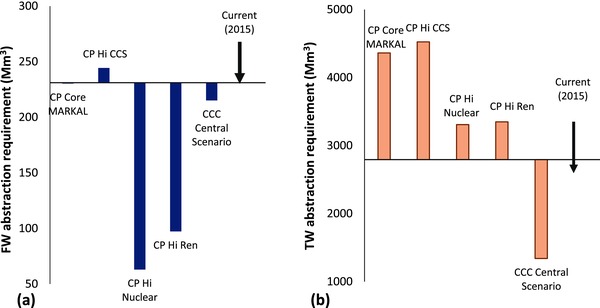
Comparison of the total current (2015) and 2030 water resource abstraction requirement for the CCC Central scenario and the UK Carbon Plan pathways (CP) (presented in ref. 12): a) Freshwater (FW) and b) Tidal water (TW). [Hi CCS = High Carbon Capture and Storage; Hi Nuclear = High Nuclear; Hi Ren = High Renewables].

**Figure** **6** presents a comparison of the total UK water resource requirement for thermal power generation and crude oil refinery associated with the CCC Central scenario and UK 2050 Carbon Plan in 2030 relative to current (2015) requirements. Overall, freshwater requirements in 2030 for the CCC Central scenario are projected to significantly reduce relative to current abstractions, and a future dominated high nuclear and renewable electricity generation technologies under the UK 2050 Carbon Plan. However, a future energy system dominated by high CCS, as in the case of the High CCS (Hi CCS) pathway under the Carbon Plan, would require significantly higher freshwater to deploy compared to the CCC Central scenario. On the other hand, the overall tidal water resource requirement of the CCC Central scenario significantly reduces relative to current requirements, and significantly lower than all future energy system pathways of the Carbon Plan in 2030. These alternative energy system pathways are described in Table S5 (Supporting Information).

## 3 Discussion

The analysis presented in this study illustrates a significant departure from the current water abstraction regime for the energy sector. Water resource abstractions are projected to change significantly by 2030 at both national and catchment levels as a direct consequence of decarbonizing the energy sector via the CCC Carbon Budgets. With the exception of the Trent basin which projects significant increase for power generation, freshwater requirements decreases across all basins. Tidal water abstraction requirements are also projected to decreases across all basins, with the exception of the Thames and Scotland basins. The significant increases in water requirement in the Trent, Thames basins, and Scotland are mainly attributable to the relatively high capacity of projected deployment of CCS technology with gas and coal for power generation, which are more water intensive.

With regard to the spatial downscaling assumptions of future energy infrastructure location used in this study, these basins have existing high capacity legacy sites of coal generation, and are in effect projected to host most of the CCS capacity. By extension more water resources will be required to deploy the projected technologies and capacities. Commensurate with the theme of decarbonization, future consumption of liquid fossil fuel, in particular gasoline and diesel for transport are projected by the CCC Carbon Budgets to significantly decrease by 2030. This decrease is projected to translate into reduced fossil fuel refining across the UK relative to current capacity. It is however, assumed that the overall percentage of refined petroleum product that is exported from the UK would remain the same as today.

The projected increase in water resource requirements associated with the deployment of the different energy technologies prescribed under the CCC Central scenario suggests that some UK catchments could face increased competition for water resources. In particular, water availability and competition challenges in the Thames basin, which is the most densely populated basin in the UK, could be further exacerbated or expected generation capacity constrained. This is due to the fact that the total water demand from all sectors in this basin currently exceeds available resource in most areas (Figure 2), thus a significant increase in water demand by the energy sector will worsen the current situation. The Trent, basin which currently hosts the highest thermal electricity generation capacity in the UK could also face increased water resource competition challenges as freshwater requirements are estimated to more than double by 2030 per the CCC Central Scenario projections. While the implications of these results suggest energy generation in the Trent Basin may become significantly water constrained by 2030 (if indeed legacy sites are redeveloped), this will depend on how the demand from other competing uses of water also changes in this part of the country. These changes notwithstanding, water resource implications of the CCC Central scenario projections is estimated to lead to decreased water resource requirements across most other river catchments in the UK, with the most significant decrease associated with freshwater abstractions in the Yorkshire Ouse basin, which hosts the highest biomass electricity generation capacity in the UK (i.e., Drax Power station). This decrease can be explained by the low overall biomass generation projection by 2030, which together with the spatial downscaling approach used in the study (mainly predicated on the location of current and legacy power stations), leads to a lower electricity generation capacity in the basin relative to current generation.

The comparison of overall UK water resource requirements between the CCC Central Scenario and the UK Carbon Plan (presented in Figure 6) highlights the critical differences with regards to water resource appropriation associated with different future energy system trajectories. Even though all the different energy system trajectories meet similar GHG emissions reduction targets, they project varying water resource requirements, and would pose different challenges. The CCC Central Scenario would lead to significant freshwater resource implications, but poses the lowest impact on tidal water resources relative to current (2015) and all the Carbon Plan pathways. The differences could be directly attributed to the deployment of relatively high capacity water intensive technologies, including high CCS and CCGT capacities in place of retiring coal power generation under the Carbon Plan. This stems from the fact that both the CCC Central scenario and the Carbon Plan pathways are predicated on similar assumptions of cooling technologies and energy system infrastructure.

The methodology and approach used in this study is intended to illustrate potential water resource implications of a long‐term national energy system projection at the catchment level using a simplified linear spatial downscaling model. This approach can be replicated in other jurisdictions for high‐level planning and decision‐making on the water‐energy nexus. More importantly, even though overall water resource impacts at the national level may be minimal, a more spatially resolved (basin‐level) analysis reveals significantly variable levels of impacts and potential generating constraints. Although most long‐term energy system planning is done at the national level, applying a similar methodology in analyzing the regional water‐energy nexus implications would provide critical insights on potential water resource challenges at the most appropriate spatial level that could inform policy decisions on where best to site future energy system infrastructure that are water intensive. This is particularly essential in countries and regions where water resource demand is uncertain, and in major river catchments which are projected to experience significantly increased water demand across all sectors due to increased population and economic growth. Conducting a water‐energy nexus assessment using similar methodologies presented in this study, alongside demand for water from other sectors would help elicit critical future challenges that energy provision in other countries could face if certain water intensive energy technologies are deployed.

However, the assumptions considered in this study have inherent uncertainties, particularly with regards to the location of future energy infrastructure. The of use of legacy sites for specific energy technologies in the future is based on the assumption of having access to the current/historical water available at the site. This may not materialize since water abstraction licensing are based on availability and environmental flow requirements. Since this study has not incorporated climate change implications on water resource availability, future variations in precipitation and river flow levels could result in the inability to deploy the projected capacities within the basins. This is particularly critical since future precipitation and river flows across the UK are projected to vary significantly, both spatially and temporarily.^10^ Thus allocating future energy generation capacity to specific basins, based on current/legacy siting may lead to inherent uncertainties regarding the feasibility of deploying projected energy technologies and capacities which essentially rely on water resource availability and abstraction licensing. It is therefore imperative that in applying the approach presented in this study to other jurisdictions, in particular rapidly expanding economies (including population) and water resource‐poor regions, the potential impacts of climate change on water resource availability are adequately considered. Nevertheless these results highlight the importance of considering water and energy interdependencies at the appropriate scale.

## 4 Conclusions and Wider Implications for Other Jurisdictions

This paper has for the first time presented the potential water resource implications of future low‐carbon UK energy system transitions of the CCC Carbon Budgets at the catchment/regional level. Additionally, the paper illustrates the overall national‐scale water resource implications of the CCC Carbon Budgets relative to current and alternative long‐term energy system pathways presented by the UK 2050 Carbon plan. It can be concluded from the results and the above discussion that while the energy system trajectory in the Carbon Budgets to 2030 projects minimal water resource impacts on a national scale, some basins in the UK could face significant water resource challenges in the future due to increased water resource requirements.

The analysis highlights the critical importance of analyzing the water‐energy nexus at a spatial resolution that captures the essential elements that characterizes water availability and abstraction licensing. However, since most long‐term national energy policies are developed at the national scale, assessing the long‐term water resource implications at the basin level, as presented in this study, becomes rather challenging. According to Khan et al.,^17^ the main challenges associated with water and energy system integration at the appropriate scales stem from the distinct spatio–temporal and physical characteristics of the systems, site‐specific complementary data availability, and the degree of model aggregation and generalization of current assessment approaches. Thus, despite the limitations discussed above, the approach used in this study presents a heuristic method that can be replicated in other jurisdictions to provide critical insights on water resource implications of nationally planned long‐term energy policies at the basin level.

The analysis presented in this study, has only focused on the water requirement for thermal electricity generation and oil refining, which represent the main current water‐energy nexus issue in the UK. However, there are other water‐energy nexus issues, which even though not critical in the UK, have significant implications in other jurisdictions, in particular water resources poor regions. These include water use in large‐scale bioenergy production (e.g., refs. 18, 19), energy use in irrigation (e.g., ref. 20), and energy use in public water supply (including pumping, treatment, and transport) (e.g., ref. 21). Within the UK, future onshore hydrocarbon exploitation (fracking) presents significant uncertainties for water resources, in particular groundwater quality.^22^, ^23^ These issues have direct socio–economic and environmental sustainability implications at both regional and basin scales. It is therefore imperative that a more holistic approach to analyzing water‐energy nexus that incorporates basin level analysis, and captures the above‐mentioned interdependencies be developed to support decision‐making at various levels of resource governance.

## 5 Experimental Section

The CCC Carbon Budgets present the long‐term blue‐print of the UK Government's GHG emissions reduction strategy across different sectors of the economy, in line with the UK Climate Change Act (2008), i.e., to reduce GHG emissions by at least 80% on 1990 levels by 2050.^1^ The main energy system trajectory of the Carbon Budgets for meeting 2050 GHG emission reduction is defined by the “Central Scenario.” The Central Scenario presents an optimized least cost estimate pathway to meeting the 2050 GHG target, while balancing other Government economy‐wide aims, including businesses competitiveness, and energy security and affordability.^24^ The emissions reduction targets associated with the Central Scenario remains the most critical aspect of meeting overall targets, as it relates to all the major sectors of the UK economy. It is therefore imperative that the projected energy system mix that leads to meeting stipulated GHG emissions reduction targets is not hampered by the availability of natural resources required for their deployment. Thus, in the context of this study, water resource availability and the sustainable appropriation thereof to support the deployment of different energy technologies across the whole energy provision value chain the Central Scenario are of utmost importance.

In order to ensure that all aspects of the projected energy mix of the CCC Central scenario are adequately captured and analyzed for their water resource implications, this study employs an integrated whole system resource accounting methodology. This method follows a similar approach presented in Konadu et al.^12^ and Schoonbaert,^25^ and involves a top‐down analysis of the interconnections and resource use accounting of the energy system. The approach maps, tracks, and estimates the water resource requirement for different sectors of the energy system supply chain, spanning indigenous primary resource extraction and production, fossil fuel refining, and thermal power generation. However, this paper considers only water use in thermal power generation and fossil fuel (crude oil) refining, which are the two main water resource abstraction sectors with potential significant implications for future water resource availability. In the UK, two main sources of water resources, fresh (surface and groundwater) and tidal water (usually brackish river sections influenced by sea tides), are licensed for public, agricultural and industrial abstraction. These sources are located inland in rivers, streams, canals, and aquifers, and are distinct from seawater, which is currently not licensed. This study analyses only fresh and tidal water resources of which over abstraction could have direct implications for aquatic ecosystems.

Three main analytical stages are considered: (1) mapping of the linkages between the Central Scenario energy system projections and water resources, and downscaling these from a national (UK) to major river catchments (in England and Wales) and at the regional level in Scotland and Northern Ireland, based on the current spatial distribution of energy system infrastructure; (2) estimation of the water requirements for the deployment of the different energy system technologies of the Central Scenario to 2030 within the major river catchments in England and Wales, and for Scotland and Northern Ireland; and (3) comparison of the output of the overall water resource requirements of the Central Scenario and the 2050 Carbon Plan pathways (based on a study by Konadu et al.^12^) at the national scale.

**Figure 7:**
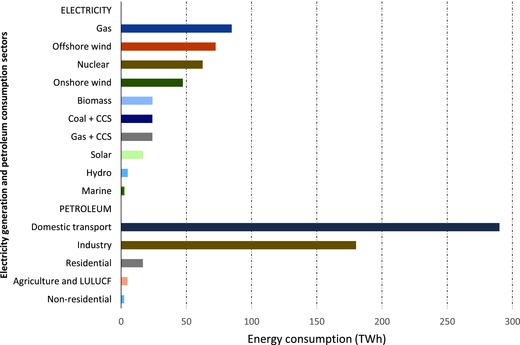
Configuration of electricity generation technologies and petroleum consumption sectors for the CCC Central Scenario in 2030.

*The Central Scenario Energy Mix and Associated Sources of Water Demand*: The energy system technology configuration and demand of the Central Scenario for 2030 is presented in **Figure** **7**. The main demands from the Central Scenario for water resources are from thermal electricity generation using gas, nuclear, biomass, gas, and coal with CCS, and petroleum products refining. The main water requirement for thermal electricity generation is associated with process steam production and cooling, whilst petroleum refining (which supplies the transport sector) requires water for process cooling. This study focuses mainly on these processes, and therefore excludes water used for sanitary services, fire protection, and miscellaneous purposes, which are relatively in small quantities.

**Figure 8:**
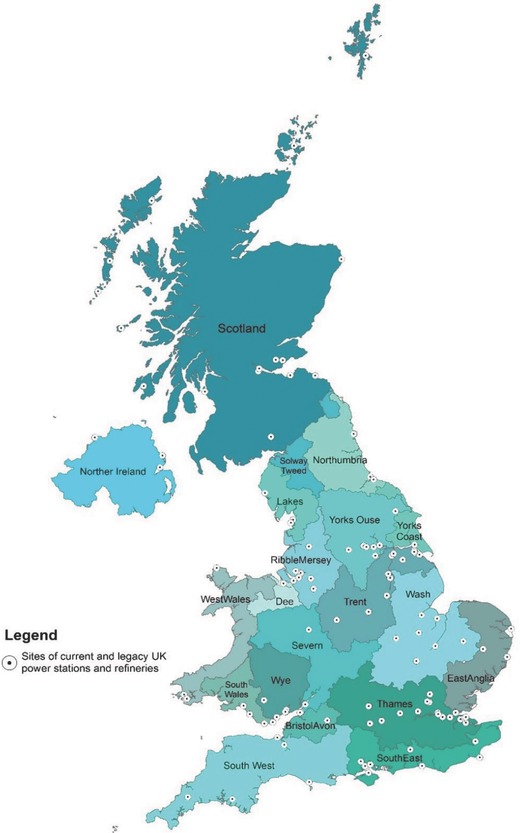
Current distribution of power stations and petroleum refinery infrastructure in the UK by fuel/technology for major river catchments (hydrological regions)/countries (Source: ref. [2,26]).

*Estimation of the Water Requirement of the CCC Central Scenario Energy Mix*: The water requirements required for the CCC Central Scenario technologies and the associated processes depend on two main factors—the cooling technology used, and the location of the energy infrastructure. The type of cooling technology deployed influences the amount of water required (demand), whereas the location of the energy infrastructure determines the source and type of water used (available supply). While current location and cooling technologies deployed in the UK for electricity generation and petroleum refining are known (**Figure** **8**), future location of energy infrastructure and by extension the source of water and the cooling technologies to be deployed for operations is difficult to determine as these are currently unspecified. This remains a critical challenge in the estimation of future water resource implications of different energy system trajectories. This is particularly so as water resource implications are best analyzed at the catchment level where data from existing water resource assessments is known and available. Hence the need to have spatially explicit long‐term energy system pathways. The Central Scenario poses this challenge as it is restricted to a national‐scale energy system pathway.

To overcome the challenge of the nonspatiality of the Central Scenario, this study uses a linear downscaling approach, which assumes that all future large‐scale energy system infrastructures will be located in the same major river catchments as today. This assumption is predicated on the fact that the major UK river catchments, which currently host large‐scale energy system infrastructure, have been assessed by the UK Environment Agency to have the capacity to provide water sustainably to support current energy provision. A significant part of energy generation infrastructure, particularly coal power and nuclear power plants, is set to be retired by 2025. Therefore, the current basins could provide brown‐field sites with sustainable water resources (based on current water abstraction licenses for electricity) support new energy infrastructure. Equation (1) shows a simplified linear model used in the downscaling of the CCC Central scenario from a national scale to major river catchments in England and Wales, and at the national level in Scotland and Northern Ireland

(1) $$C_{\mathrm{Trs}}=\%C_{\mathrm{Trc}}*C_{\mathrm{Tns}}$$

Where: *C*_Trs_ = Capacity (*C*) of technology (T) in river catchment/country (r) for the CCC scenario (s).

%*C*_Trc_ = percentage of the Capacity (*C*) of current (c) technology (T) in river catchment/country (r).

*C*_Tns_ = projected national (n) Capacity (*C*) of technology (T) for the CCC scenario (s).

The output of the spatial downscaling of the CCC central scenario is then used to estimate the associated future water resource requirements in each river catchment/country. The water resource requirement estimation follows an approach presented in Konadu et al.,^12^ which combines the capacity of different power generation, and oil refining technologies with associated water use per unit of energy (electricity or petroleum) output to estimate the overall water requirement for each river catchment/country, i.e., with respect to Scotland and Northern Ireland. This approach presents as a linear resource accounting model illustrated in Equation (2)

(2) $$W_{\mathrm{rs}}=\overset{}{\underset{}{\overset{n}{\underset{WT\;=\;\;i}{\sum }}}}\;C_{\mathrm{Ti}}\;W_{\mathrm{Ti}}$$

Where: *W*_rs_ = Total water requirement in river catchment/country (r) for the CCC scenario (s).

*C*_Ti_ = Capacity (*C*) of a particular technology (Ti) in river catchment/country (r).

*W*_Ti_ = Water requirement per unit of energy produced by a particular technology (Ti).

While the CCC Central scenario projects the different energy technologies and primary fuel at the national level, which can be spatially downscaled using the above approach, the cooling technologies that would be deployed are not specified. This present a critical question of what cooling technologies would be deployed alongside different technologies in the future? While this question cannot be definitively answered here, a sensitivity analysis approach has been taken to assess the potential impact of different choices of cooling technologies on water resource requirement for the energy system. This involves the estimation of the water requirement for two scenarios of cooling technologies: (1) a baseline scenario which assumes that current cooling technologies and water sources associated with current energy technologies are be maintained; (2) a reduced water withdrawal (abstraction) scenario which assumes that all energy technologies except nuclear will use a combination of evaporative, hybrid and dry cooling technologies. The characteristics and abstractions volumes (in l/TWh) of these cooling technologies are described in ref. 11 and Table S1 (Supporting Information).

*Comparison of Water Requirements of the CCC Central Scenario and the 2050 Carbon Plan*: The final step of the analysis involves the assessment of the overall sustainability of the water resource appropriation associated with the CCC Central Scenario relative to current^24^ and the UK 2050 Carbon Plan pathways.^9^ First, we present a spatial comparison of the current (2015) levels of fresh and tidal water abstraction requirements, and water resource availability across all major river catchments/hydrological regions (country) of the CCC's CCRA2 updated projections for water availability for the UK.^16^ The CCRA2 water availability data presents the annual water supply‐demand balance of the various water resource management zones, and is illustrated as a percentage demand of the available resources. This comparison is done to show the current implication of the energy system for water resources within different basins. This is followed by a comparison of the output of the water requirement analysis in 2030 with the projected energy mix of the CCC Central Scenario with the current levels (2015) of abstraction for all major river catchments/country in the UK. This comparison illustrates the potential stress or otherwise on water resources within these major river catchments under the low‐carbon energy system transition projections of the CCC.

The final assessment in this study involves the comparison of the catchment scale output with the water resource requirement projected for the UK 2050 Carbon Plan pathways in 2030 on a national scale. The Carbon Plan, like the CCC Central Scenario, presents alternative low‐carbon energy system pathways to 2050 that achieve the UK's 2050 GHG emissions target. The comparison therefore seeks to answer the question of whether water resource requirements could provide an additional sustainability measure for making decisions on different long‐term low‐carbon energy system trajectories. The analysis uses the output of the water resource requirement associated with the Carbon Plan pathways presented in Konadu et al.,^12^ which is based on similar resource accounting methodology and assumptions used in this study.

## Conflict of Interest

The authors declare no conflict of interest.

## Supporting information

Supplementary
