# Supplementary material for: Catchment Level Water Resource Constraints on UK Policies for Low‐Carbon Energy System Transitions by 2030
Source: Glob Chall. 2017 May 11;1(5):1700006. doi: 10.1002/gch2.201700006 (PMC6607168; doi:10.1002/gch2.201700006)
Supplement: Supplementary file 1 — Supplementary [file GCH2-1-1700006-s001.pdf]

Copyright WILEY-VCH Verlag GmbH & Co. KGaA, 69469 Weinheim, Germany, 2017.

Supporting Information

for *Global Challenges*, DOI: 10.1002/gch2.201700006

Catchment Level Water Resource Constraints on UK Policies for Low-Carbon Energy System Transitions by 2030

D. Dennis Konadu and Richard A. Fenner*

Supplementary information

SI Table 1 : Characteristics of different power generation cooling system (based on Byers et al., 2014)

Cooling system	Description
Once through (open loop)	Heat is removed through transfer to a running water source (can be direct or indirect).
Closed (re-circulatory)	Heat is removed to the air by recirculating water cooled in ponds or under cooling towers that may be fan-assisted or natural draught.
Air-cooled	Heat is removed by air circulation via fans and radiators. A setup that can operate without water.
Hybrid d	Cooling towers that can operate both with and without cooling water – either combining a wet/dry cooling tower, or a dry then wet system in series.

SI Table 2: Water abstraction and consumption factors for different electricity generation technology and primary fuel

**Abstraction (m^3/TWh)**	**Coal/Biomass**	**Gas**	**Oil**	**Nuclear**
Once through freshwater cooling	118.50 **(1,2)**	49.30 **(2)**	134.40 **(2)**	164.40 **(1,2)**
Once through freshwater cooling + CCS	220.00 **(4)**	90.00 **(4)**	-	-
Once through saline cooling (freshwater component)	0.22 **(5)**	0.011 **(5)**	0.011 **(5)**	0.011 **(5)**
Wet-Tower cooling	1.82 **(1,2,3)**	0.98 **(2)**	2.08 **(2)**	3.90 **(1,2,3)**
Wet-Tower Cooling + CCS	4.29 **(1,3)**	1.82 **(1,3)**	-	-
Hybrid	1.30 **(1,2,3)**	0.60 **(2)**	0.70 **(2)**	-
Hybrid + CCS	2.80 **(1,3)**	1.20 **(1,3)**	-	-
Air (Dry) cooling	0.22 **(5)**	0.011 **(5)**	0.011 **(5)**	-

(1) Macknick et al., 2011; (2) EPRI, 2002; (3) NETL, 2009; (4) Tzimas, 2011; (5) Williams and Simmons, BP., 2013

SI Table 3: Water requirements and consumption for oil refining

**Oil Refining**		Source
**water abstraction**	**m^3/TWh**	Williams and Simonds, BP (2013)
Once through cooling	0.95	
Process	0.01	
Steam	0.02	
Total water withdrawal for oil refining	0.98	

SI Table 4: Water requirement for current (2015) and 2030 for power generation and oil refining in the UK under the Committee on Climate Change Carbon Budgets

Basins/Regions	Power FW 2015 (Mm^3)	Power TW 2015 (Mm^3)	Power CCC FW 2030 (Mm^3)	Power CCC TW 2030 (Mm^3)	Refining FW 2015 (Mm^3)	Refining TW 2015 (Mm^3)	Refining CCC FW 2030 (Mm^3)	Refining CCC TW 2030 (Mm^3)
Bristol Avon	0.0	0.0	0.0	2.1	0.0	0.0	0.0	0.0
Dee	0.0	7.0	0.0	1.9	0.0	0.0	0.0	0.0
East Anglia	0.1	127.1	0.0	8.9	0.0	0.0	0.0	0.0
Lakes	0.9	0.0	0.1	0.0	0.0	0.0	0.0	0.0
Northern Ireland	0.6	123.4	0.0	25.9	0.0	0.0	0.0	0.0
Northumbria	1.1	0.3	3.2	2.4	0.0	0.0	0.0	0.0
Ribble Mersey	5.7	19.0	1.8	2.0	5.4	174.9	2.8	91.9
Scotland	0.2	0.0	0.2	132.5	3.5	118.4	1.9	62.1
Severn	0.0	4.5	0.9	0.7	0.0	0.0	0.0	0.0
Solway Tweed	0.0	0.0	0.0	0.0	0.0	0.0	0.0	0.0
South East	0.1	276.7	0.0	125.3	4.9	159.7	2.6	83.9
South Wales	1.9	3.1	0.3	0.2	0.0	0.0	0.0	0.0
South West	0.1	0.0	0.0	0.0	0.0	0.0	0.0	0.0
Thames	16.4	4.4	5.3	320.4	0.0	0.0	0.0	0.0
Trent	82.3	635.6	169.8	98.9	7.7	248.9	4.0	130.8
Wash	4.5	0.0	1.2	0.0	0.0	0.0	0.0	0.0
West Wales	0.2	686.4	0.0	144.3	6.0	194.4	3.2	101.9
Wye	0.1	2.7	0.0	0.8	0.0	0.0	0.0	0.0
Yorkshire Coast	0.0	7.2	0.0	1.9	0.0	0.0	0.0	0.0
Yorkshire Ouse	89.5	0.0	17.5	0.0	0.0	0.0	0.0	0.0

SI Table 5: Description of the different Carbon Plan pathways considered in this study (based on Byers et al., 2014 and Konadu et al., 2015)

Carbon Plan pathway	Description
Core MARKAL	This includes a combination of technologies and resources that minimise system cost by 2050, estimated by the UK MARKAL energy model
High Renewables (Hi Ren)	This is pathway considers higher renewables and more energy efficiency. Investment and innovation in renewables and storage driven by high fossil fuel prices and global commitment to tackling climate change. Mix of wind, solar and marine renewables, backed up by gas.
High Nuclear (Hi Nuclear)	This pathway deploys higher nuclear generation and assumes less energy efficiency. Nuclear dominates and CCS is assumed not to be commercially viable. Gas meets peak demands and energy efficiency is low. Heat and transport are largely electrified.
High Carbon Capture and Storage (Hi CCS)	This pathway assumes a commercial availability and deployment of CCS carbon capture and storage (CCS) for electricity generation and industry fuelled by high levels of natural gas imports due to low fossil fuel prices and extensive shale gas. Involves negative emissions through Biomass + CCS.

References

1. US Department of Energy (2006) Energy demands on water resources; Report to Congress on the interdependency of energy and water. Available at http://www.sandia.gov/energy-water/docs/121-RptToCongress-EWwEIAcomments-FINAL.pdf (Accessed 20/06/2014)

2. Macknick, J., Newmark, R., Heath, G., Hallett, K.C., 2011. A Review of Operational Water Consumption and Withdrawal Factors for Electricity Generating Technologies. National Renewable Energy Laboratory, USA. , Available at http://www.nrel.gov/docs/fy11osti/50900.pdf. (Accessed 23/05/2014)

3. Electric Power Research Institute Inc. (EPRI) (2002). Water & Sustainability (Volume 3): U.S. Water Consumption for Power Production – The Next Half Century. Palo Alto, CA, USA, Available at http://www.epri.com/abstracts/Pages/ProductAbstract.aspx?ProductId=000000000001006786 (Accessed 23/05/2014)

4. National Energy Technology Laboratory (NETL) (2009). Water Requirements for Existing and Emerging Thermoelectric Plant Technologies. U.S. Department of Energy, National Energy Technology Laboratory, USA. , Available at

http://www.netl.doe.gov/File%20Library/Research/Energy%20Analysis/Publications/DOE-NETL-402-080108-WaterRequirements.pdf (Accessed 23/05/2014)

5. Tzimas, 2011; Tzimas, E., 2011. Sustainable or Not? Impacts and Uncertainties of Low-Carbon Energy Technologies on Water. European Commission, Joint Research Centre, Seville, Spain. , Available at https://ec.europa.eu/jrc/sites/default/files/jrc_aaas2011_energy_water_tzimas.pdf (Accessed 23/05/2014)
6. Pan L, Liu P, Ma L, Li Zheng (2012). A supply chain based assessment of water issues in the coal industry in China. Energy Policy 48, 93-102
7. Williams E. D. and Simmons J. E., BP (2013): Water in the energy industry. An introduction. BP International Ltd. Available at www.bp.com/energysustainabilitychallenge (Accessed 23/08/214)
8. Byers, E. A., Hall, J. W., & Amezaga, J. M. (2014). Electricity generation and cooling water use: UK pathways to 2050. Global Environmental Change, 25, 16-30.
9. Konadu, D. D., Mourão, Z. S., Allwood, J. M., Richards, K. S., Kopec, G. M., McMahon, R. A., & Fenner, R. A. (2015a). Not all low-carbon energy pathways are environmentally “no-regrets” options. Global Environmental Change, 35, 379-390.
